# The Health Crisis of Tuberculosis in Prisons Extends beyond the Prison Walls

**DOI:** 10.1371/journal.pmed.1000383

**Published:** 2010-12-21

**Authors:** The PLoS Medicine Editors

## Abstract

The *PLoS Medicine* editors discuss the persistent problem of tuberculosis in prisons around the world and how it affects the health of inmates and the community outside.

The scourge of tuberculosis (TB) in prisons remains a persistent problem; rates among inmates remain much higher—from 5 to up to 50 times—than those of national averages across both the developed and the developing world [1]–[5], and the situation may be worsening in some regions. In Europe and Central Asian countries, recent research has shown a clear relationship between the rate of growth of prison populations and the increased incidence of TB and multidrug-resistant TB [6]. And at a recent meeting on TB/HIV in the WHO European Region that focused on integrating treatment of these diseases [7], Diane Havlir, chair of the Stop TB Partnership's TB/HIV Working Group, opened proceedings by asking “What about prisons? Mandatory hospitalization for drug-susceptible TB? We need a debate about these things and to make improvements.”

Some important gaps in our understanding of TB in prisons exist: it's still unclear how much TB there is within prisons and what fraction of TB in the general population is contributed by disease in incarcerated groups. An article in this week's *PLoS Medicine* helps bridge that gap. Iacapo Baussano and colleagues systematically reviewed all the available published evidence on incident latent tuberculosis infection (LTBI) and TB in prisons worldwide, and by doing so were able to estimate the proportion of TB in national populations that is due to TB in prisons. The most striking finding is that, although it is clear that the rate of TB is higher in prisons than in the community, even after a thorough search and rigorous analysis of the evidence, it is not possible to give accurate estimates of the increased incidence of TB in prisons over community rates [8].

The paucity of reliable research evidence on TB in prisons found in this review should spur a renewed research effort. Such research is desperately needed to better inform policies to protect not only the health of people that are incarcerated, but also the health of the community outside.

Prisons provide ideal conditions for TB transmission. The bacterium causing TB is distributed in tiny liquid droplets that are produced when someone with active TB coughs, sneezes, spits, or speaks, enabling one person to infect many others. Therefore, the risk of TB being transmitted in settings in which people are in close contact—as in prisons and hospitals—is particularly high. Numerous other factors, such as poor health services frequently encountered in prisons, poor nutrition, drug addiction, and the presence of other diseases, such as HIV infection, are risk factors that predispose imprisoned people to a high risk of TB incidence.

Obstacles to tackling TB in prisons include inadequate infection control measures, conflicts between infection control measures and prisoner segregation (according to crime and length of sentence), lack of adequate medical facilities, lack of resources (including prison staff), and the low priority that policymakers give to health care within the prison system. Roberts and colleagues outlined how 20 jails in the US implemented measures to control TB and prevent new infections [9]. Even in this high-income setting, only four of the 20 prisons had conducted risk assessments for TB transmission, 11 had monitored tuberculin skin test conversions of inmates and staff, and only nine of the 20 prisons had policies to offer HIV counseling and testing to those with TB—an alarming situation given that HIV infection is the single largest cofactor for development of TB infection.

The new systematic review published in *PLoS Medicine* provides much-needed evidence for policymakers, including WHO, to renew their efforts to tackle TB in prisons. There is ample guidance available on how best to control and prevent TB in prisons [10],[11] and many academics, health workers, and campaigners have called for the intensive screening of high-risk populations, including prisoners, to be introduced [12] as part of an integrated strategy to reduce global incidence of TB. Future research areas uncovered by this systematic review include measuring the impact of conditions in prisons on TB transmission and assessing the population attributable risk of prison-to-community spread.

On 13 October 2010, the Global Plan to Stop TB 2011–2015 was launched by the Stop Tuberculosis Partnership (a coalition of more than 400 organizations worldwide) with the aim of halving TB mortality and prevalence rates by 2015 compared with a 1990 baseline [13]. One objective to achieving this aim is to “Ensure early diagnosis of all TB cases,” including vulnerable populations such as prisoners. The publication of this systematic review marks a shift from considering the incidence of TB in each prison population to considering the massive global impact of tuberculosis in prisons.

## Abbreviations

LTBI: latent tuberculosis infection
TB: tuberculosis
